# Evolution of the pathogenic mold *Aspergillus fumigatus* on high copper levels identifies novel resistance genes

**DOI:** 10.1128/msphere.00253-24

**Published:** 2024-05-30

**Authors:** Mariana Handelman, Zohar Meir, Yona Shadkchan, Ammar Abo Kandil, Orin Amano, Melani Mariscal, Manuel Sánchez López-Berges, Nir Osherov

**Affiliations:** 1 Department of Clinical Microbiology and Immunology, Sackler School of Medicine, Tel-Aviv University, Tel-Aviv, Israel; 2 Departamento de Genética, Campus de Excelencia Internacional Agroalimentario ceiA3, Universidad de Córdoba, Córdoba, Spain; University of Georgia, Athens, Georgia, USA

**Keywords:** *Aspergillus fumigatus*, laboratory evolution, Cu resistance, Pma1, Gcs1, Cpa1

## Abstract

*Aspergillus fumigatus* is the leading cause of severe mold infections in immunocompromised patients. This common fungus possesses innate attributes that allow it to evade the immune system, including its ability to survive the high copper (Cu) levels in phagosomes. Our previous work has revealed that under high Cu levels, the *A. fumigatus* transcription factor AceA is activated, inducing the expression of the copper exporter CrpA to expel excess Cu. To identify additional elements in Cu resistance, we evolved *A. fumigatus* wild-type and mutant Δ*aceA* or Δ*crpA* strains under increasing Cu concentrations. Sequencing of the resultant resistant strains identified both shared and unique evolutionary pathways to resistance. Reintroduction of three of the most common mutations in genes encoding Pma1 (plasma membrane H^+^-ATPase), Gcs1 (glutamate cysteine-ligase), and Cpa1 (carbamoyl-phosphate synthetase), alone and in combination, into wild-type *A. fumigatus* confirmed their additive role in conferring Cu resistance. Detailed analysis indicated that the *pma1* mutation L424I preserves Pma1 H^+^-ATPase activity under high Cu concentrations and that the *cpa1* mutation A37V confers a survival advantage to conidia in the presence of Cu. Interestingly, simultaneous mutations of all three genes did not alter virulence in infected mice. Our work has identified novel Cu-resistance pathways and provides an evolutionary approach for dissecting the molecular basis of *A. fumigatus* adaptation to diverse environmental challenges.

**IMPORTANCE:**

*Aspergillus fumigatus* is the most common mold infecting patients with weakened immunity. Infection is caused by the inhalation of mold spores into the lungs and is often fatal. In healthy individuals, spores are engulfed by lung immune cells and destroyed by a combination of enzymes, oxidants, and high levels of copper. However, the mold can protect itself by pumping out excess copper with specific transporters. Here, we evolved *A. fumigatus* under high copper levels and identified new genetic mutations that help it resist the toxic effects of copper. We studied how these mutations affect the mold’s ability to resist copper and how they impact its ability to cause disease. This is the first such study in a pathogenic mold, and it gives us a better understanding of how it manages to bypass our body’s defenses during an infection.

## INTRODUCTION

Copper (Cu) is a biologically essential metal, serving as an important co-factor in electron transfer reactions such as mitochondrial ATP generation, the reduction and utilization of nitrogen, the uptake of iron, and the generation of the pigment melanin (1). However, Cu is a double-edged sword: at high concentrations, it is toxic, displacing other metal co-factors from their apoenzymes and generating reactive oxygen species (ROS) through Fenton chemistry. Living organisms have evolved to maintain non-toxic levels of intracellular Cu using dedicated importers and chaperones for Cu uptake and transfer, as well as exporters and detoxifying enzymes to protect the cell if Cu levels become too high (2, 3).

In fungi, overcoming excessive Cu is crucial in medical settings. Pathogenic fungi encounter toxic Cu levels during infection, following phagocytosis into macrophages and neutrophils. Phagosomes express high levels of the Cu transporter Ctr1 to generate toxic levels of Cu, which kill the ingested pathogen (4). This ability is reduced or lost in immunocompromised patients who are, therefore, at great risk of contracting lethal fungal infections. Fungi respond to excess Cu by blocking its uptake and by activating a Cu-binding transcription factor that activates the expression of Cu-sequestering metallothioneins, superoxide-degrading superoxide dismutases, and Cu-efflux pumps. Inactivation of these response genes reduces the ability of human fungal pathogens to survive within the phagolysosome and attenuates virulence in infected animals (1).

*Aspergillus fumigatus* is a common saprophytic mold and a significant pathogen in immunocompromised individuals (5). It produces abundant airborne asexual spores (conidia) that, when inhaled, lead to a spectrum of diseases ranging from allergic bronchopulmonary aspergillosis to invasive pulmonary aspergillosis (IPA) (5). The endogenous alveolar macrophages constitute the first line of immunological defense against IPA. These cells primarily eliminate *A. fumigatus* through ingestion into the phagolysosome, where *A. fumigatus* encounters oxidative and enzymatic stress and toxic levels of Cu (6). In response, *A. fumigatus* activates the Cu-sensing transcription factor AceA, triggering the upregulation of the Cu-efflux pump CrpA, which reduces the toxic intracellular levels of Cu. Deletion of either *aceA* or *crpA* results in heightened sensitivity to Cu and oxidative stress *in vitro*, increased susceptibility to macrophage-mediated killing, and reduced virulence in murine infection models (6–10).

*In vitro* evolution is a powerful tool that has been successfully applied to discover novel mechanisms of antifungal resistance (11). In this study, we used a stepwise evolutionary approach and whole-genome sequencing (WGS) to identify novel genes conferring Cu resistance in *A. fumigatus*. Repeatedly occurring mutations within the genes encoding Pma1 (plasma membrane H^+^-ATPase), Gcs1 (glutamate cysteine-ligase), and Cpa1 (carbamoyl-phosphate synthetase) were reintroduced singly and in combination into the parental Cu-susceptible strain and analyzed. Our findings reveal previously unknown genetic pathways contributing to Cu resistance in the clinically significant human pathogenic fungus, *A. fumigatus*.

## MATERIALS AND METHODS

### Media and strains

Strains were grown on YAG agar plates (0.5% yeast extract, 1% dextrose, 0.01 M MgSO_4_, trace elements solution, vitamin mix, and 1.5% agar) for 48–72 h at 37°C. Conidia were collected in 0.02% Tween solution. Minimal inhibitory concentration (MIC) experiments were performed in YAG broth (0.5% yeast extract, 1% dextrose, 0.01 M MgSO_4_, trace elements solution, and vitamin mix). *A. fumigatus* transformed protoplasts were plated on YPGS agar plates (2% yeast extract, 0.5% peptone, 2% D-glucose, 1 M sucrose, 1.5% agar for plates or 0.7% for top agar, and 250 µg/mL hygromycin, pH = 6). MM agar plates contained 70 mM NaNO_3_, 1% (wt/vol) glucose, 12 mM potassium phosphate pH 6.8, 4 mM MgSO_4_, 7 mM KCl, and 0.1% (vol/vol) trace elements solution, 1.5% (wt/vol) agar. The strains used in this study are specified in Table S1.

### Generation and verification of strains used in this study

*pma1-* and *gcs1*-mutated strains were generated and selected as described previously using the pTEL-hph^R^ plasmid for the selection of transformants (12) (see Tables S2 to S4; Fig. S1 to S3). *cpa1-*mutated strains were generated using the same transformation method and selected with the *hph* resistance cassette. Transformed strains were sequenced to verify the introduction of the mutation in the target gene.

### Cu susceptibility testing

MIC was determined by broth microdilution according to CLSI M38-A2 methodology, except that RPMI MOPS liquid medium was replaced with YAG. Briefly, Cu solution was added to YAG broth and loaded into 96-well plates, conidia were diluted to 5 × 10^4^ conidia/mL and were loaded into the wells. The plates were incubated at 37°C for 48 h, and then the lowest concentration inhibiting fungal growth (observed by an inverted light microscope) was set as the MIC. Droplet susceptibility assay was performed by inoculation of 10^4^, 10^3^, 10^2^, or 10 conidia in 10 µL of 0.02% Tween on the surface of YAG agar plates containing different concentrations of Cu or other stressors and incubation for 48–72 h at 37°C.

### Evolution of Cu-resistant strains

Conidia (2 × 10^7^) of CEA10 were plated on 4 mM Cu YAG plates in four replicates (lineages) and another control plate without Cu. Conidia (2 × 10^7^) from Δ*aceA* or Δ*crpA* strains were plated on 1 mM Cu YAG plates in three replicates (lineages) and another control plate without Cu. After 72–96 h, all the conidia from each Cu plate were collected separately and transferred to a plate containing a higher Cu concentration. Control conidia were transferred in parallel to YAG plates without Cu. This stepwise evolution was performed until no further growth or conidiation occurred at the highest Cu concentration. Single isolates from the highest Cu concentration were purified and repeatedly passaged on YAG agar, and their resistance was quantified by standard broth microdilution and droplet assays. To ensure that *aceA* and *crpA* deletion strains remained pure, the respective gene deletion of each lineage was tested every few generations and in the last generation by PCR.

### Whole-genome sequencing and analysis

Genomic DNA from all final isolates was extracted and validated in an agarose gel. DNA concentration and purity were assessed by NanoDrop and the Qubit RNA/DNA HS Assay Kit (Thermo Fisher Scientific). Libraries were prepared with the NEBNext Ultra II FS DNA Library Prep Kit for Illumina (BioLabs Inc.), and their purity was assessed with TapeStation. Libraries were loaded into the NextSeq 500/550 Mid Output Kit v2.5 (300 Cycles) (Illumina), and the sequencing was performed using the NextSeq 500 machine. The results were analyzed at the Core facility of the Weizmann Institute (Rehovot, Israel).

### Determination of gene expression by RT-qPCR

Strains were grown on YAG agar plates for 72 h, and 6 × 10^7^ spores were collected and inoculated in 150 mL of YAG broth in 500 mL flasks. Flasks were incubated under shaking for 22 h at 37°C, then a 0.5 MIC concentration of copper was added to three 25 mL flasks for 2 h, and three 25 mL flasks were left untreated (a total of six flasks per strain). Mycelium was collected, lyophilized, and crushed; RNA was extracted with the QIAGEN RNeasy Plant Mini Kit. RNA concentration was assessed using NanoDrop (Thermo Fisher Scientific), then equal amounts of RNA from each sample were converted to cDNA with the Verso cDNA Synthesis Kit (Thermo Fisher Scientific). Equal amounts of cDNA (based on RNA amounts) from each sample were loaded into Applied Biosystems MicroAmp Optical 96-Well plates with Applied Biosystems Fast SYBR Green Master Mix and primer for either *β-tubulin* (housekeeping control gene), *aceA, crpA, pma1, gcs1*, or *cpa1*.

2^-ΔΔCt^ analysis was performed. Statistical analysis was performed with two-way ANOVA with Tukey’s multiple comparison test.

### Determination of Pma1 H^+^-ATPase activity

For the determination of the activity of the plasma membrane H^+^-ATPase Pma1 in *A. fumigatus*, 5 × 10^6^ conidia/mL of each strain were inoculated on ammonium tartrate minimal medium with trace elements (TE) lacking copper (MM + TE^-Cu^) and incubated at 37°C and 200 rpm for 20 h (−Cu). Afterward, cultures were supplemented, or not, with 10 µM (+Cu) or 10 mM (hCu) CuSO_4_ and incubated for five more minutes. Germlings were rapidly harvested by filtration through a nylon filter (mesh size 10 µm) and flash-frozen in liquid nitrogen. Plasma membrane fractions were obtained as previously described (13, 14). Protein concentration was determined with the DC Protein Assay (Bio-Rad) by reading absorbance at 750 nm in a spectrofluorometer (Infinite M200 PRO, TECAN Life Sciences). The specific Pma1 H^+^-ATPase activity was calculated as previously described (13). Briefly, the residual activity obtained after adding the specific Pma1 inhibitor diethylstilbestrol was subtracted from the total activity (methanol solvent) and expressed in mmol/min/g protein assayed. The results represent the average and standard deviation of three independent biological experiments with three technical replicates each. Statistical analysis was performed according to two-way ANOVA and Bonferroni test.

### Murine model of invasive aspergillosis

Six-week-old ICR female mice were immunocompromised with two subcutaneous injections of 300 mg cortisone acetate/kg of body weight, administered 3 days before and on the day of infection. Anesthesia was induced with an intraperitoneal injection of 100/10 mg ketamine + xylazine/kg of body weight, and the mice were intranasally infected with 5 × 10^5^ dormant conidia/mouse, in 20 µL of 0.2% Tween 20 in saline solution (0.9% [wt/vol] NaCl) (10 µL in each nostril). Sacrifice endpoints included a drop of greater than 15% in body weight or signs of acute distress. The mice were monitored for 21 days, and the results were analyzed by the log-rank test for Kaplan–Meyer survival curves in GraphPad Prism software.

## RESULTS

### The evolution of *A. fumigatus* on high levels of Cu generates resistant strains

**Fig 1:**
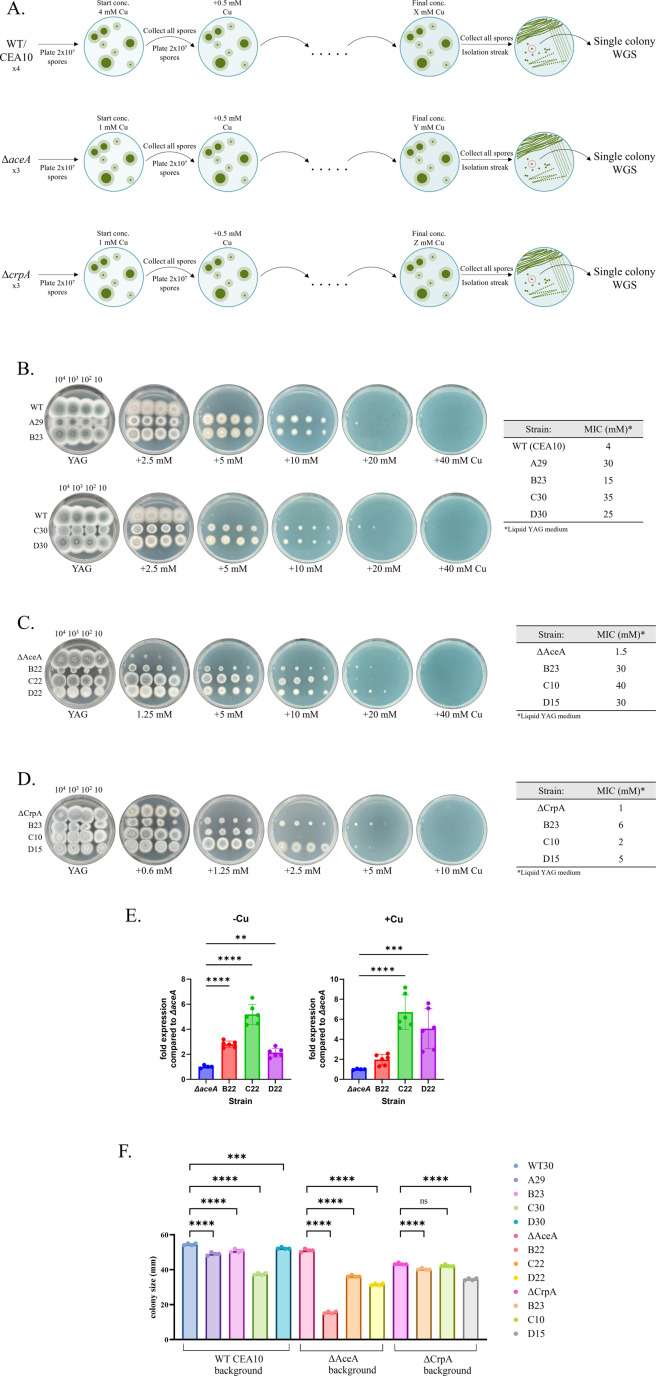
Stepwise evolution of *A. fumigatus* under elevated Cu levels. (**A**) Stepwise evolution was performed by passaging four independent lines of *A. fumigatus* WT/CEA10 or three lines of Δ*aceA* and Δ*crpA* Cu-sensitive mutant strains on YAG agar plates containing increasing concentrations of Cu. Single colonies growing at the highest viable Cu concentration were selected for WGS and phenotypic characterization. Comparison between (**B**) the WT and evolved Cu-resistant strains A29, B23, C30, and B30, (**C**) Δ*aceA* and evolved Cu-resistant strains B22, C22, and D22, and (**D**) Δ*crpA* and evolved Cu-resistant strains B23, C10, and D15. The left image shows point inoculation on YAG agar plates (10^4^–10 colony-forming units/point inoculation) with increasing Cu concentrations, while the right table presents the determination of their MICs in liquid YAG. (**E**) Increased *crpA* mRNA expression in the evolved strains B22, C22, and D22 compared to the parental Δ*aceA* strain (****P < 0.0001 and ***P < 0.0001, by one-way ANOVA). (**F**) Radial growth of all evolved strains. A total of 300 conidia were point inoculated in quadruplicate on YAG agar plates, incubated for 3 days at 37°C, and measured for colony size. ****P < 0.0001 according to one-way ANOVA and Holm-Šídák’s multiple comparisons test.

To reveal novel mechanisms of Cu resistance in *A. fumigatus*, we performed stepwise evolution under increasing Cu concentrations. Four plates of parental wild-type (WT) and three plates of Δ*aceA* and Δ*crpA* strains were serially passaged on YAG agar containing progressively higher Cu concentrations (Fig. 1). We hypothesized that subjecting both the Δ*aceA* and Δ*crpA* strains, in addition to the WT, to evolutionary pressures may reveal resistance mutations that operate independently of the extensively characterized AceA/CrpA axis of Cu resistance. Selection began at the subinhibitory concentrations of 4 mM Cu for the WT strain and 1 mM for the Cu-sensitive Δ*aceA* and Δ*crpA* strains and increased by 0.25–1 mM Cu at each step (Fig. 1A). A control strain was simultaneously passaged in the absence of Cu. The evolutionary process was terminated at the passage where the Cu concentration inhibited conidiation or growth entirely. Then, a single colony from the highest viable Cu concentration was selected for phenotypic and WGS analysis. Subsequent passaging of these isolated resistant strains without Cu demonstrated that they maintained stable resistance and displayed a consistent phenotype (data not shown). Strains A29, B23, C30, and D30 evolved from the WT strain, showed a four- to eightfold increase in Cu resistance on liquid YAG and a three- to fourfold increase in Cu resistance on YAG agar (Fig. 1B). Strains B22, C22, and D22 evolved from the Δ*aceA* strain, displayed a ≥20-fold increase in Cu resistance on liquid YAG and an eightfold increase in Cu resistance on YAG agar. (Fig. 1C). Strains B23, C10, and D15 evolved from the Δ*crpA* strain, exhibited a two- to sixfold increase in Cu resistance on liquid YAG and a two- to eightfold increase in Cu resistance on YAG agar (Fig. 1D). Remarkably, the copper-resistant mutants evolved in the WT and Δ*aceA* strains displayed significantly higher Cu MIC values (15–40 mM) compared to those evolved in the Δ*crpA* strain (2–6 mM). This discrepancy might be due to the fact that the WT- and Δ*aceA*-evolved strains contain, in contrast to Δ*crpA*, a functional *crpA* gene (the major copper resistance-mediating gene), whose transcriptional activation depends on AceA. Therefore, to test if Cu evolution reactivated *crpA* expression in Δ*aceA*, we used RT-qPCR to compare *crpA* expression in the mutant strains evolved from Δ*aceA*. Strains Δ*aceA,* B22, C22, and D22 were cultured in liquid YAG for 22 h at 37°C and exposed to 0.5 MIC Cu for 2 h, followed by RNA extraction and qPCR analysis (Fig. 1E). We found that the basal expression of *crpA* in the evolved Δ*aceA*-background strains B22, C22, and D22 was two- to fourfold higher compared to the control unevolved Δ*aceA*-background strain (Fig. 1E, –Cu). Moreover, upon Cu induction, the expression of *crpA* in strains C22 and D22 increased by four to sixfold compared to the control unevolved Δ*aceA*-background strain (Fig. 1E, + Cu). These findings strongly suggest that under Cu selection, these strains have evolved to express CrpA in an AceA-independent manner.

Adaptation to stressful conditions frequently entails a fitness cost upon the removal of these conditions. To test this, we measured the radial growth of the evolved mutants in the absence of Cu. In the absence of Cu, all evolved strains except Δ*crpA* C10 displayed a decrease in radial growth rates, indicating that adaptation to elevated Cu levels is associated with a fitness cost in its absence (Fig. 1F).

### WGS of the evolved Cu-resistant *A. fumigatus* strains identifies recurrent mutations in *pma1*, *gcs1,* and *cpa1*

To identify mutations conferring Cu resistance, we performed WGS and sequence analysis of the evolved strains and compared them to the control strains passaged in parallel in the absence of Cu. The published sequence of the WT-CEA10 genome was used as a scaffold. Overall, the evolved Cu-resistant strains displayed limited coding mutations (between two and six genes per strain, Table 1), duplications, and deletions (Table S5). We focused our analysis on coding mutations in genes that exhibited alterations across multiple, independently evolved strains, as we reasoned that they were most likely to confer resistance. *pma1* (AFUB_041460) was mutated in 8 of the 10 evolved strains. *pma1* is an essential gene encoding the central plasma membrane H^+^-ATPase, which generates the proton gradient that drives the active transport of nutrients (15). *gcs1* (AFUB_035300), also named *gshA*, encoding a glutamate cysteine-ligase, catalyzing the first step in the biosynthesis of the antioxidant glutathione (GSH) (16), and *cpa1* (AFUB_054340), encoding a carbamoyl-phosphate synthetase, involved in an early step of arginine biosynthesis (17), were mutated in all four WT-evolved strains.

**TABLE 1:** Mutations identified by WGS in the Cu-evolved strains

Strain	Mutations*^a^*
WT A29	**Gcs1** FD481-482del; **Cpa1** A37V; AFUB_065670/Prx1 D104G; AFUB_016190/Ysh1 V162A; AFUB_055510 V163A
WT B23	**Pma1** L424I; **Gcs1** FD481-482del; **Cpa1** A37V; AFUB_055510 V163A
WT C30	**Pma1** L424I; **Gcs1** FD481-482del; **Cpa1** A37V; AFUB_021700/Hse1 G326Stop; AFUB_081890/Myo1 D820N; AFUB_055510 V163A
WT D30	**Pma1** L424I; **Gcs1** FD481-482del; **Cpa1** A37V; AFUB_069070 L101P; AFUB_055510 V163A
ΔaceA B22	**Pma1** V186del; AFUB_095920/E2 ubiquitin-conjugating K9N
ΔaceA C22	**Pma1** A414G; AFUB_071830 Y331stop; AFUB_088200/PIB1 ubiquitin ligase G192R
ΔaceA D22	AFUB_024580/SltB L77F; AFUB_035240/Psd2 Q597stop; AFUB_021070 /Png1 F186S; AFUB_078980 S251L
ΔcrpA B23	**Pma1** V186del; CtrB/AFUB_040930 T142M; AFUB_034400 R464K; AFUB_055940/Ssk1 R690W; AFUB_059540/PkcA R50W; AFUB_081980/CoaT I255N
ΔcrpA C10	**Pma1** N158D; CtrB T142M; AFUB_011550/DscA D528G; AFUB_042970 S515P; AFUB_090650 D528G
ΔcrpA D15	**Pma1** L176P; CtrB T142M

^*a*^ Key mutations referred to in this study are marked in bold.

### Mutations in *pma1* and *gcs1* confer Cu resistance in *A. fumigatus*

**Fig 2:**
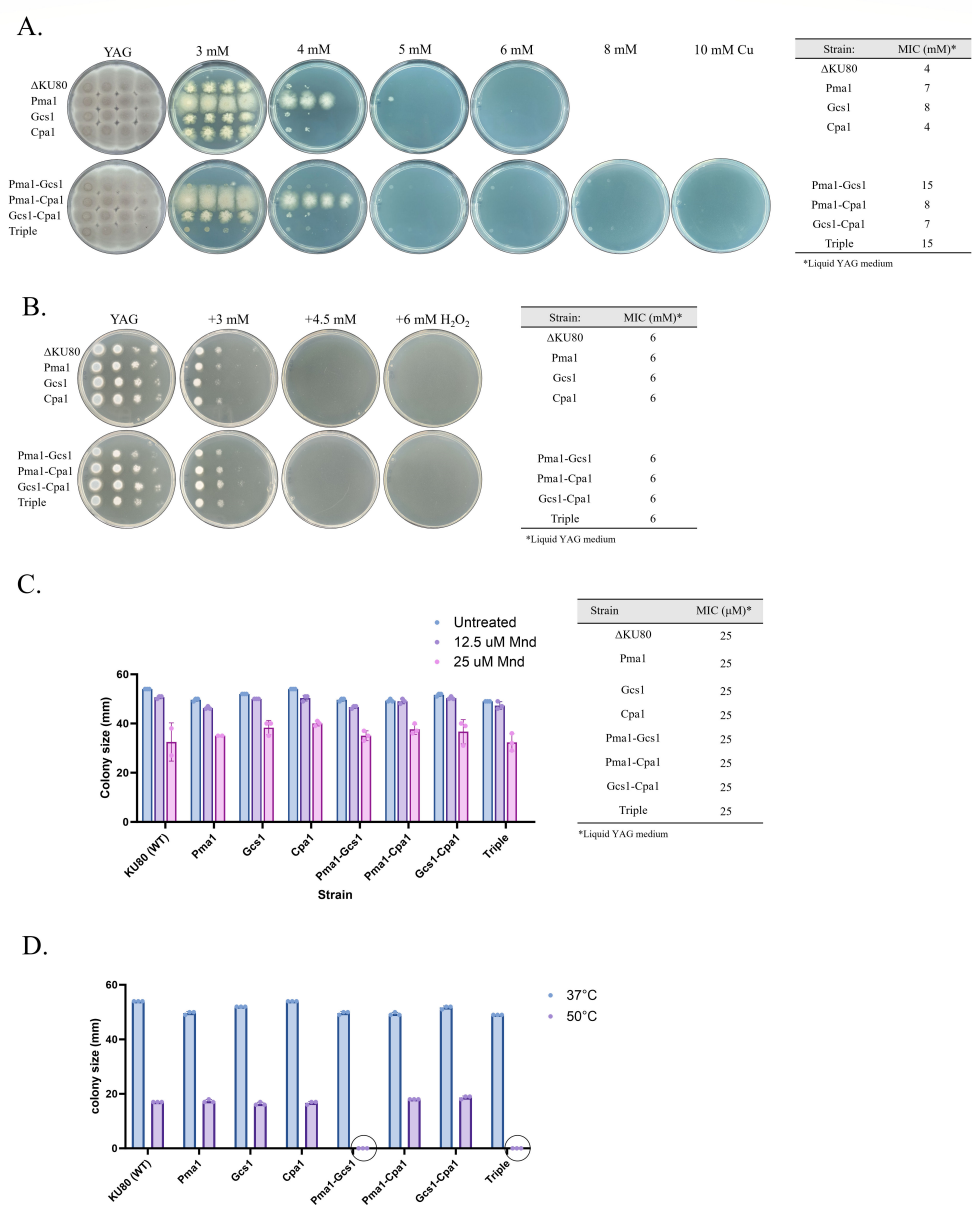
Contribution of *pma1*, *gcs1,* and *cpa1* mutations to Cu resistance in *A. fumigatus*. Single Pma1, Gcs1, Cpa1; double Pma1-Gcs1, Pma1-Cpa1, Gcs1-Cpa1; and triple Pma1-Gcs1-Cpa1 mutant strains were generated by the introduction of mutated *pma1* (L424I), *gcs1* (FD481-482del), and *cpa1* (A37V) into the ΔKU80 (WT) strain. These mutants were assessed for their resistance to (**A**) Cu, (**B**) H_2_O_2_, and (**C**) menadione (Mnd) or (**D**) growth at 50°C. For **panel A**, the left image shows point inoculation on YAG agar plates (10^4^–10 CFU/point inoculation) for 3 days at 37°C, with increasing Cu concentrations, while the right table presents the determination of Cu MICs in liquid YAG. For panel **B**, the left image shows point inoculation on YAG agar plates (10^4^–10 CFU/point inoculation) for 24 h at 37°C, with increasing H_2_O_2_ concentrations, while the right table presents the determination of H_2_O_2_ MICs in liquid YAG. For panel **C**, the left image shows radial colony growth on YAG agar plates (300 conidia per point inoculation) for 3 days at 37°C, with increasing Mnd concentrations, while the right table presents the determination of Mnd MICs in liquid YAG. (**D**) Radial growth on YAG agar plates at 37°C and 50°C for 3 days.

To assess the contribution of mutated *pma1* (L424I), *gcs1* (FD481-482del), and *cpa1* (A37V) to Cu resistance, the mutated genes were introduced, alone and in combination, into a *KU80* null derivative of the WT strain (ΔKU80) (18) to generate single Pma1, Gcs1, Cpa1; double Pma1-Gcs1, Pma1-Cpa1, Gcs1-Cpa1; and triple Pma1-Gcs1-Cpa1 mutant strains. We used CRISPR-Cas9 to excise the WT version of the gene and replace it with the corresponding mutated version, as previously described (12). We generated two to four independent PCR-positive transformants for each strain, all of which exhibited a similar phenotype (see full details for strain construction and verification in the supplemental material). To evaluate Cu sensitivity, strains were either point inoculated and grown for 3 days at 37°C on YAG agar plates containing increasing Cu concentrations or grown for 2 days at 37°C in liquid YAG and subsequently assessed for growth inhibition (Fig. 2). A single insertion of *pma1* (L424I) in strain Pma1 resulted in an almost twofold increase in Cu-MIC in liquid YAG medium compared to the control ΔKU80 (WT) strain, as well as a noticeable increase in resistance on YAG agar (Fig. 2A). A single insertion of *gcs1* (FD481-482del) in strain Gcs1 resulted in a twofold increase in Cu-MIC in liquid YAG medium relative to the control ΔKU80 (WT) strain, but only a weak increase in resistance on YAG agar. In strains Pma1-Gcs1 and triple (Pma1-Gcs1-Cpa1), the introduction of both *pma1* (L424I) and *gcs1* (FD481-482del) led to an additive 3.75-fold increase in Cu-MIC in liquid YAG medium compared to the control KU80 (WT) strain and a twofold increase in resistance on YAG agar (Fig. 2A). Cu resistance was unaffected by the introduction of *cpa1* (A37V), either alone or in combination with *pma1* (L424I) and *gcs1* (FD481-482del) (Fig. 2A).

Cu exposure generates ROS and induces cellular oxidative stress (1). To determine whether the mutations in *pma1*, *gcs1,* or *cpa1* contribute to Cu resistance by enhancing the capacity to withstand oxidative stress, we conducted experiments where strains were cultured in both liquid and solid YAG media containing increasing concentrations of H_2_O_2_ or menadione (Mnd), a compound that induces oxidative stress. Surprisingly, we observed no discernible differences in either H_2_O_2_ or Mnd sensitivity among the tested strains, whether they were grown on agar (MIC_H2O2_ = 6 mM; MIC_Mnd_ = 50 µM for all strains) (Fig. 2B) or in liquid medium (MIC_H2O2_ = 6 mM; MIC_Mnd_ = 25 µM for all strains) (Fig. 2C). These results collectively suggest that the mutations in *pma1* (L424I) and *gcs1* (FD481-482del) confer Cu resistance through mechanisms that do not involve enhanced resistance to oxidative stress.

### Fitness costs associated with the *pma1*, *gcs1,* and *cpa1* mutations

Mutations that confer resistance to a specific stressor are frequently linked to a fitness cost or benefit when exposed to other stressors. To assess the influence of the *pma1*, *gcs1,* and *cpa1* mutations to affect the fitness landscape in *A. fumigatus*, strains ΔKU80, Pma1, Gcs1, Cpa1, Pma1-Gcs1, Pma1-Cpa1, Gcs1-Cpa1, and triple (Pma1-Gcs1-Cpa1) were point inoculated on YAG agar plates for 3 days under various stress conditions. They included low or high pH (4, 5, and 8.5), high salt (NaCl 1.5 M), hypoxia (1% O_2_), reductive conditions (DTT 1 mM), membrane stress (SDS 5 µg/mL), or heat (50°C). Radial growth measurements revealed significant growth differences between the strains only when grown at 50°C (Fig. 2D; Fig. S4). Strains Pma1-Gcs1 and triple (Pma1-Gcs1-Cpa1) were unable to grow at 50°C, indicating that the combination of mutations in *pma1* (L424I) and *gcs1* (FD481-482del) is lethal at this temperature.

### The *pma1* (L424I) mutation allows the maintenance of Pma1 H^+^-ATPase activity under toxic Cu concentrations

**Fig 3:**
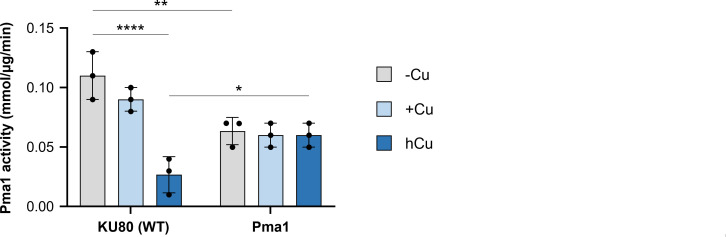
Toxic Cu concentrations inhibit Pma1 but not Pma1(L424I) activity. Pma1 ATPase activity was assayed in total membrane fractions isolated from the indicated strains grown in ammonium tartrate minimal medium −Cu (20 h in copper depletion), +Cu (20 h in copper depletion and then supplemented with 10 µM CuSO_4_ for 5 min), and hCu (20 h in copper depletion and then supplemented with 10 mM CuSO_4_ for 5 min). ****P < 0.0001, **P < 0.002, and *P < 0.033 according to two-way ANOVA and Bonferroni test. Bars represent standard deviations from three independent biological experiments with three technical replicates each.

High levels of Cu disrupt membrane integrity, possibly inhibiting the function of essential membrane transporters such as Pma1. We hypothesized that at high Cu concentrations, the *pma1* (L424I) mutation increases Pma1 H^+^-ATPase stability, thus retaining sufficient activity to partially overcome membrane damage. To test this hypothesis, we determined Pma1 H^+^-ATPase activity in *A. fumigatus* wild-type strain (KU80 WT) and in the Pma1 mutant, growing both strains under copper deficiency (−Cu) and shifting them to a non-toxic (+Cu) or a toxic (hCu) copper concentration. Indeed, shifting the wild-type strain from −Cu to hCu conditions significantly reduced Pma1 activity, while the shift to +Cu conditions did not have a substantial effect. Importantly, although Pma1 activity was significantly lower in the Pma1 mutant relative to the wild-type strain in −Cu conditions, it remained stable in both +Cu and hCu conditions (Fig. 3). Collectively, our results reveal that the *pma1*(L424I) mutation reduces Pma1 activity in −Cu or +Cu conditions, but that this cost is reversed under hCu conditions where *pma1*(L424I) retains more than twice the activity of wild-type Pma1.

### Expression of *gcs1* is induced by Cu

**Fig 4:**
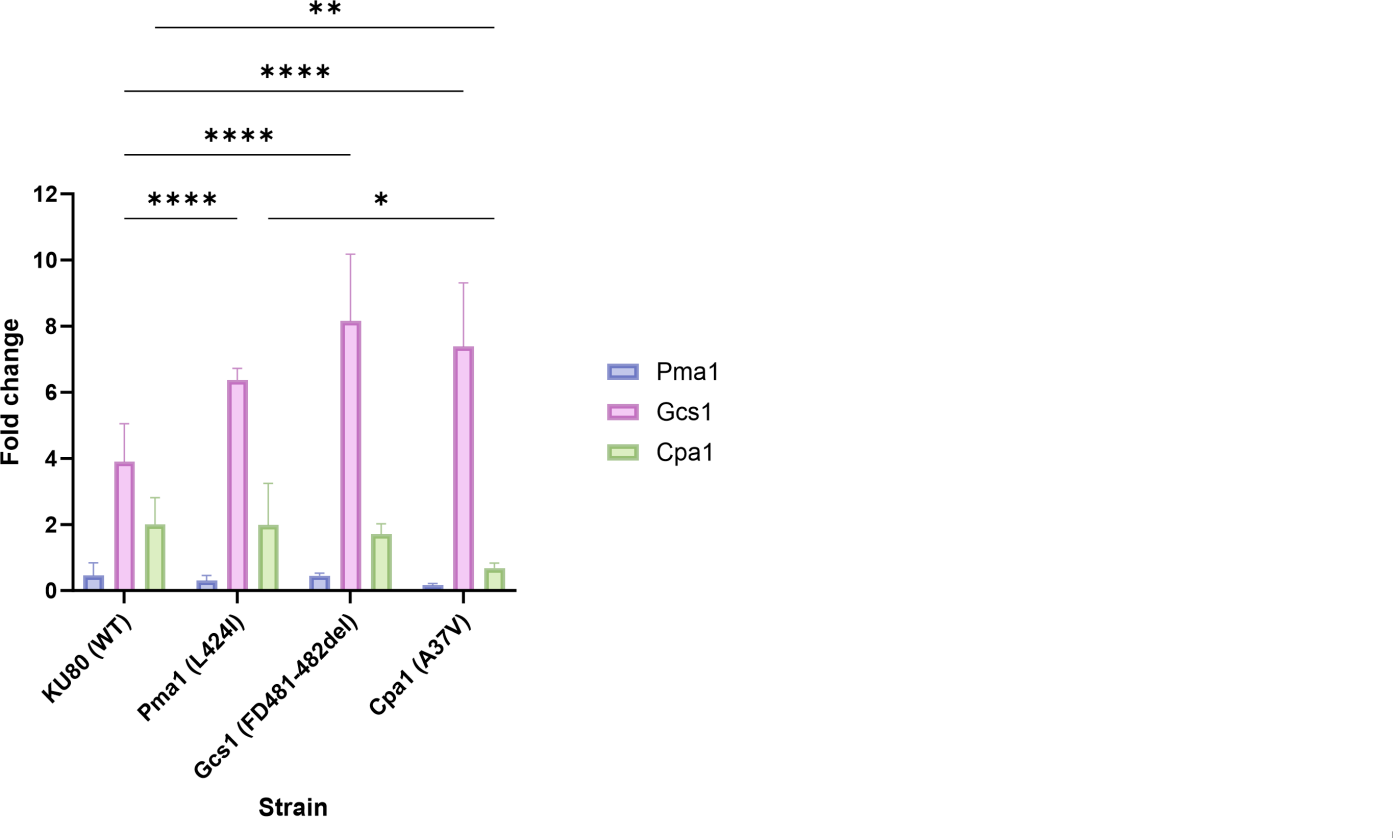
Differential *pma1*, *gcs1,* and *cpa1* expression in WT and mutant strains. Fold expression calculated as 2^-ΔΔCT^ of *pma1*, *gcs1,* and *cpa1* in WT, Pma1, Gcs1, and Cpa1 strains following exposure to Cu (treated versus untreated), (****P < 0.0001, **P = 0.007, and *P = 0.011 according to two-way ANOVA and Tukey’s multiple comparisons test). Bars represent standard deviations from three independent biological experiments with three technical replicates each.

We compared *pma1*, *gcs1,* and *cpa1* mRNA levels between the Pma1, Gcs1, and Cpa1 mutant strains and the ΔKU80 (WT) strain. Strains were grown in liquid YAG for 22 h at 37°C and exposed to sublethal Cu for 2 h, followed by RNA extraction and qPCR analysis (Fig. 4). We found that Cu induced a fourfold increase in *gcs1* expression in the ΔKU80 (WT) strain. Cu-induced *gcs1* expression in the Pma1, Gcs1, and Cpa1 mutant strains was significantly increased relative to the ΔKU80 (WT) strain.

### Mutated *cpa1* (A37V) confers a conidial survival advantage in the presence of Cu

Cpa1 catalyzes an early step in arginine biosynthesis. *cpa1* deletion leads to arginine auxotrophy in *Saccharomyces cerevisiae*, while *cpa1* overexpression leads to 5-flucytosine resistance in *Colletotrichum gloeosporioides* (17, 19). We tested whether the *cpa1* A37V mutation affects the ability of the Cpa1 mutant strain to grow in the absence of arginine or in the presence of flucytosine. We observed no difference in growth between Cpa1 and the ΔKU80 (WT) strain (Fig. S5), which suggests that the mutation does not block Cpa1 activity.

**Fig 5:**
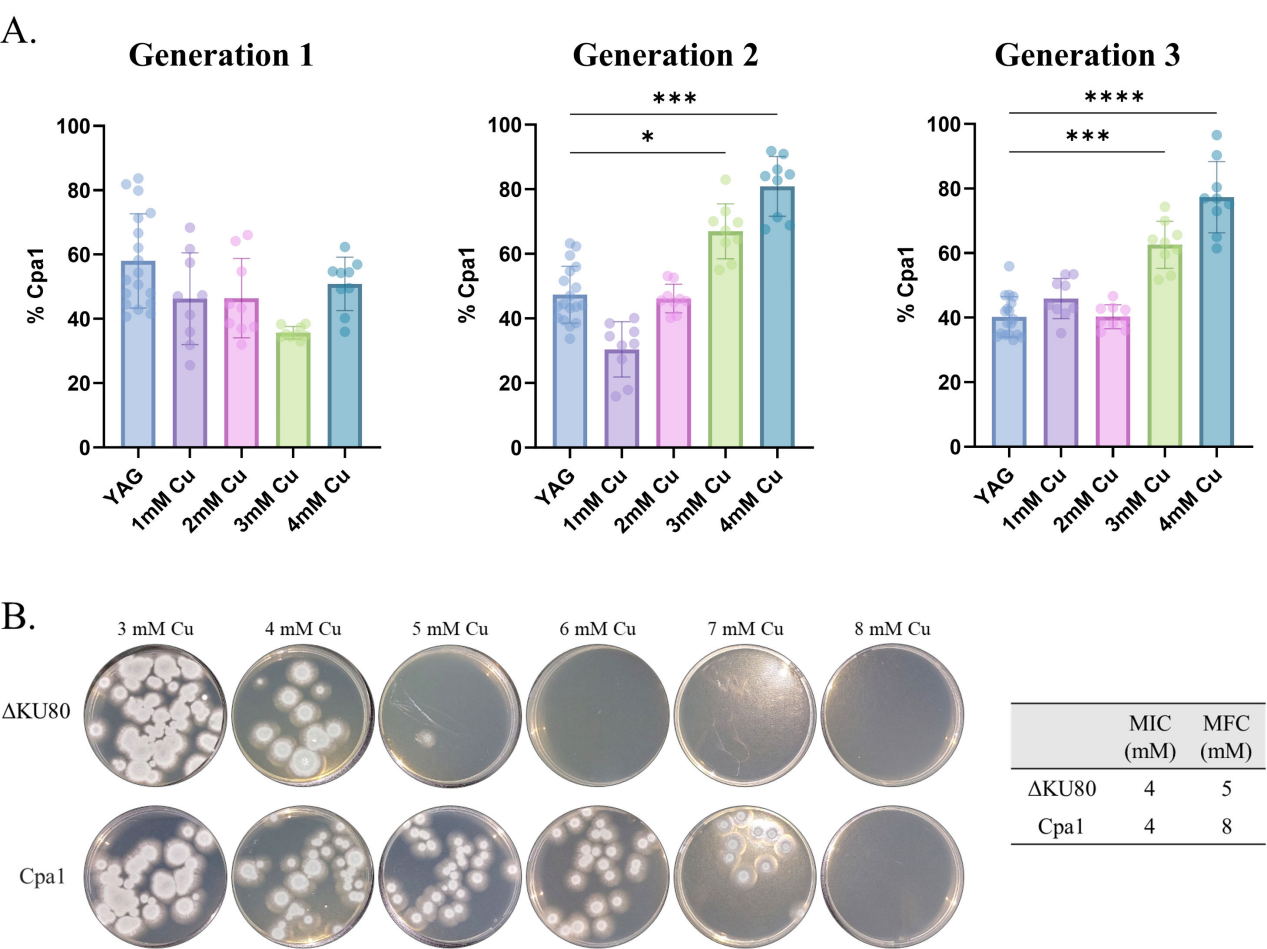
Fitness advantage of the Cpa1 mutant in the presence of Cu. (**A**) WT and Cpa1 conidia were mixed at a 1:1 ratio and grown in competition under increasing Cu for up to three generations. The percentage of Cpa1 CFUs was calculated based on their ability to grow in the presence of hygromycin, unlike the WT strain. ****P < 0.0001, **P < 0.01, and *P < 0.05 according to the one-way ANOVA Kruskal-Wallis and Dunn’s multiple comparisons test. (**B**) Determination of Cu MICs and MFCs of WT and Cpa1 strains grown in liquid YAG for 48 h at 37°C (table on the right). The plates on the left show the determination of the MFCs of WT and Cpa1 strains.

We then investigated the possibility that the *cpa1* A37V mutation enhances the survival of the Cpa1 mutant strain in the presence of Cu. We mixed freshly harvested ΔKU80 (WT) and Cpa1 conidia at a 1:1 ratio and cultured them under increasing Cu concentrations for up to three generations. For each generation, we quantified the ratio between the number of conidia generated by the ΔKU80 (WT) and Cpa1 strains through colony-forming-unit (CFU) enumeration on YAG ± hygromycin (see Materials and Methods) (Fig. 5). We observed that after two to three generations of co-cultivation in the presence of 3 and 4 mM Cu, the percentage of Cpa1 colonies versus the ΔKU80 (WT) increased significantly (Fig. 5A). We hypothesized that the increased fitness of the Cpa1 mutant strain is due to higher conidial survival in the presence of Cu. To test this, we incubated 4 × 10^3^ conidia from the ΔKU80 (WT) and Cpa1 mutant strains for 48 h at 37°C in liquid YAG containing increasing concentrations of Cu. Notably, both strains exhibited no conidial germination at 4 mM and higher Cu concentrations, establishing an MIC of 4 mM. However, when conidia from the wells containing 3–8 mM Cu were collected and plated on YAG agar (without Cu) to determine the survival by colony-forming-unit enumeration, we found that Cpa1 conidia demonstrated enhanced survivability, even at Cu concentrations of up to 7 mM, resulting in a minimal fungicidal concentration (MFC) of 8 mM, in contrast to the ΔKU80 (WT), which displayed reduced survivability at Cu concentrations of 4–5 mM (MFC = 5 mM) (Fig. 5B). In summary, these results suggest that while the Cpa1 mutation does not increase Cu resistance as measured by growth (Fig. 2A), it does increase the viability of germinating conidia under high levels of Cu, thus conferring a selective fitness advantage that is evident after several generations of co-cultivation with the ΔKU80 (WT) strain.

### *pma1* (L424I), *gcs1* (FD481-482del), and *cpa1* (A37V) mutations do not affect fungal virulence

**Fig 6:**
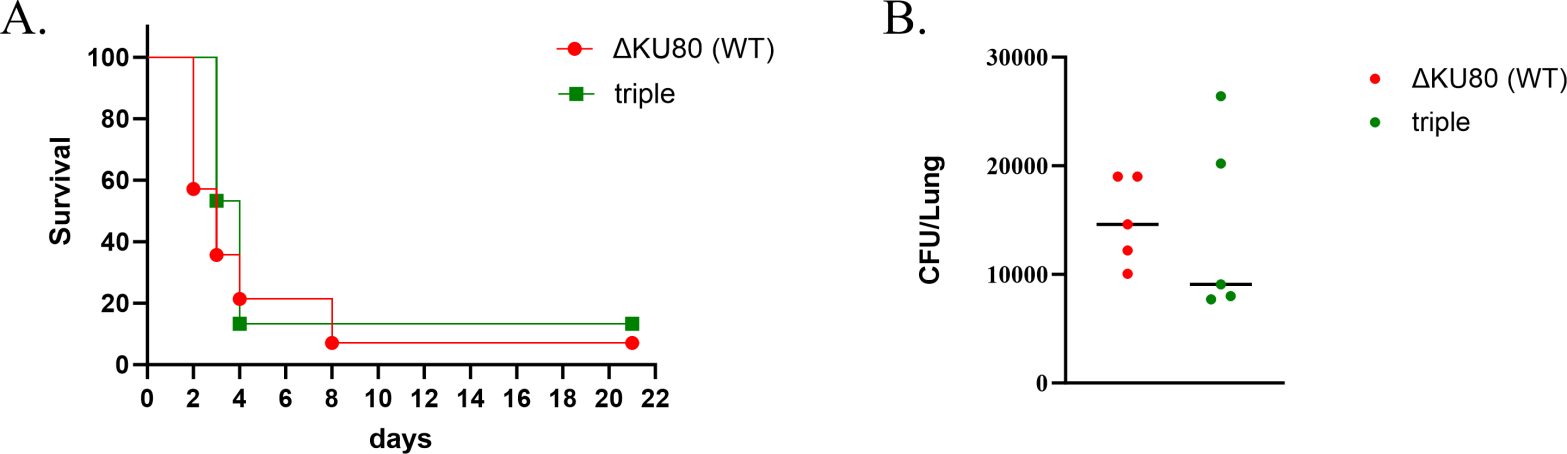
Effect of *pma1* (L424I), *gcs1* (FD481-482del), and *cpa1* (A37V) mutations on fungal virulence. Cortisone-acetate immunocompromised mice were infected intranasally with Δ*KU80* (WT) or triple Pma1-Gcs1-Cpa1 strain conidia. (**A**) Survival plot of mice during 21 days of infection. (**B**) Lung fungal load after 48 h of infection.

We previously demonstrated that deletion of the Cu exporter CrpA results in Cu sensitivity and reduced virulence in infected mice (6). Given that the triple Pma1-Gcs1-Cpa1 mutant is more resistant to Cu, we hypothesized that it would display increased virulence in mice. To test this, we used a pulmonary infection model in immunocompromised mice. Two groups of 15 mice were infected with ΔKU80 (WT)and triple mutant strains, respectively. The mice were immunocompromised and infected intranasally with *A. fumigatus* conidia as described in Materials and Methods. Mouse survival was monitored for 21 days. Surprisingly, the survival curve revealed no difference in virulence between the triple Pma1-Gcs1-Cpa1 strain relative to the ΔKU80 (WT) strain (*P* = 0.2234) (Fig. 6A). To determine whether the lung fungal load was altered in the triple Pma1-Gcs1-Cpa1 mutant strain, mice (*n* = 5) were infected as described above and sacrificed after 48 h. Their lungs were homogenized, and aliquots were spread on YAG agar plates with antibiotics. CFU counts after incubation showed no statistically significant differences in fungal load from the lungs of the triple Pma1-Gcs1-Cpa1 strain compared to the ΔKU80 (WT) strain (*P* = 0.8744) (Fig. 6B). In conclusion, the introduction of *pma1* (L424I), *gcs1* (FD481-482del), and *cpa1* (A37V) mutations does not affect virulence and fungal load in a murine model of *A. fumigatus* lung infection.

## DISCUSSION

This study investigates the genomic alterations in *A. fumigatus* WT, Δ*aceA,* and Δ*crpA* strains evolved under increasing levels of Cu. It assesses the effect of mutations in key genes encoding Pma1 (plasma membrane H^+^-ATPase), Gcs1 (glutamate cysteine-ligase), and Cpa1 (carbamoyl-phosphate synthetase) on Cu resistance.

### Experimental evolution of fungal Cu resistance

Experimental evolution of Cu resistance in fungi was previously only documented in *Saccharomyces cerevisiae*. Resistance was driven by a complex evolutionary process, which included gene amplification of the copper metallothionein protein CUP1 (20, 21), chromosomal aneuploidy, and multiple mutations in the vacuolar transporter genes VTC1 and VTC4; the plasma membrane H^+^-ATPase PMA1; and MAM3, a protein required for normal mitochondrial morphology (21). In pathogenic fungi, experimental evolution has primarily focused on the development of antifungal resistance (11, 22). This study represents the first investigation into the evolution of Cu resistance in a significant pathogenic filamentous fungus. It is also the first to compare the genomic alterations occurring in a wild-type strain with those in mutants (Δ*aceA*, Δ*crpA*) lacking key Cu resistance genes.

### Resistance to Cu evolved in distinct patterns

Our study primarily analyzed three key genes: *pma1* (mutated at least once across all backgrounds), *gcs1*, and *cpa1* (mutated in all WT strains). Introducing all three mutant gene versions into the WT strain led to the acquisition of approximately half of the resistance observed in the original evolved WT strains when grown in a liquid medium containing Cu. This is probably because of additional coding and intergenic mutations, deletions, and duplications in the original evolved strains that likely also increased resistance but were not further analyzed. Notably, each strain in each background evolved along a unique evolutionary trajectory. This intricate pattern of resistance acquisition likely reflects the complex nature of Cu in cellular functions, where it is essential at low levels but toxic through various relatively non-specific pathways at high levels (1). Consequently, there are many potential pathways for acquiring resistance.

### The *A. fumigatus pma1* mutation L424I stabilizes Pma1 H^+^-ATPase activity under high Cu levels

*pma1*, encoding the plasma membrane H^+^-ATPase, is responsible for transporting protons from the cytoplasm to the extracellular space. This process is crucial for maintaining intracellular pH and ion homeostasis, forming a transmembrane electrochemical potential, and facilitating nutrient transport and the expulsion of secondary metabolites (15). In *S. cerevisiae*, Cu-induced lipid peroxidation leads to a decline in plasma membrane lipid order and membrane disruption. Yeast counteracts the Cu-induced dissipation of the proton-motive force and the decreased intracellular pH by stimulating Pma1 activity (23). Our study demonstrates that in *A. fumigatus*, the *pma1* mutation L424I enables the maintenance of ATPase activity at high Cu concentrations, resulting in an approximately twofold increase in Cu resistance in the liquid medium.

Pma1 exhibits a characteristic topology featuring 10 membrane-spanning elements and 3 distinct cytoplasmic domains: an actuator domain, a nucleotide-binding domain, and a phosphorylation domain (24). The *pma1* mutation L424I is situated in the cytosolic phosphorylation domain, which is crucial for Pma1 activation, and is adjacent to the catalytic phosphorylatable residue D427. A neighboring Pma1 mutation, A414G, was also found in the Δ*aceA* D22-evolved mutant. In yeast, mutations of the homologous residues A365F and L375A result in reduced ATP hydrolysis and a decreased ATP Km (25). In agreement, our findings indicate that the *pma1* L424I mutation decreases ATP hydrolysis in the absence of Cu. However, unlike the wild-type Pma1, this activity is maintained under high Cu conditions. We also identified another cluster of Pma1 mutations (N158D/ΔcrpA C10, L176P/ΔcrpA D15, and V186del/ΔcrpA B23 and ΔaceA B22) within the second transmembrane domain (N158D) and adjacent actuator domain (L176P and V186del). The actuator domain undergoes a conformational change during ATP hydrolysis, allowing the transport of protons to the outside of the cell membrane (24). The precise effects of these additional mutations on Pma1 function remain to be investigated in future research.

### The *gcs1* FD481-482del mutation does not confer increased protection against oxidative stress

*gcs1*, encoding glutamate cysteine ligase, catalyzes the ATP-dependent condensation of cysteine and glutamate. This forms the dipeptide gamma-glutamylcysteine, the first and rate limiting step in GSH production, a key cellular antioxidant. In yeast, deletion of the Gcs1 homolog, GSH1, results in glutathione auxotrophy, reduced resistance to oxidative stress, and mitochondrial instability (26, 27). In *A. fumigatus*, the deletion of *gcs1* leads to glutathione auxotrophy, GSH depletion, and an iron starvation response (16). The *gcs1* FD481-482del mutation we studied does not lead to glutathione auxotrophy in the evolved or reconstituted Gcs1 strain (not shown).

The *gcs1* FD481-482del mutation, which we identified in all four evolved WT strains, led to a twofold increase in Cu resistance in a liquid medium. We hypothesized that this mutation increases Gcs1 activity, enhancing protection against oxidative stress induced by Cu exposure. However, we observed no elevation in GSH levels in the *gcs1* FD481-482del mutant (Fig. S6) or increased resistance to the oxidizing agents H_2_O_2_ or menadione.

### The *cpa1* (A37V) mutation provides a conidial survival advantage under elevated Cu levels

*cpa1*, encoding the small subunit of carbamoyl-phosphate synthetase, catalyzes the ATP-dependent deamination of glutamine to carbamoyl phosphate, representing a crucial initial stage in arginine biosynthesis. Arginine is essential in diverse biological processes, including protein biosynthesis, nitrogen metabolism, urea and nitric oxide (NO) biosynthesis, and serves as a potent antioxidant (28, 29). The *cpa1* mutation we identified (A37V) is situated upstream of the glutaminase catalytic domain and has not been previously described. Our findings reveal that Cu resistance, as assessed by growth, remained unaffected by the introduction of *cpa1* (A37V), either alone or in combination with *pma1* (L424I) and *gcs1* (FD481-482del). In contrast, we found that the *cpa1* (A37V) mutation enhances conidial survival in the presence of Cu, resulting in a fitness advantage relative to WT conidia under high Cu. A possible interpretation is that conidia from the Cpa1 strain contain elevated concentrations of arginine, which counteracts Cu-induced oxidative stress and enhances conidial survival under high Cu.

### *pma1* (L424I), *gcs1* (FD481-482del), and *cpa1* (A37V) mutations do not affect fungal virulence

Cu is used by phagocytic cells to kill *A. fumigatus* during infection. The mature phagolysosome accumulates copper at high concentrations of tens to hundreds of micromolars (30). Mutant strains of *A. fumigatus* that exhibit hypersensitivity to Cu demonstrate reduced virulence in murine models of invasive aspergillosis (6). We thus reasoned that Cu-resistant strains of *A. fumigatus* strains might demonstrate increased virulence during infection. Surprisingly, however, infection with the Cu-resistant triple mutant strain containing the *pma1* (L424I), *gcs1* (FD481-482del), and *cpa1* (A37V) mutations did not reveal any significant difference in virulence or fungal load compared to the control WT strain. This observation suggests two possibilities: either the phagolysosome accumulates levels of Cu that are sufficient to kill the triple mutant strain or other phagolysosomal antimicrobial mechanisms such as acidification, hydrolytic enzymes, antimicrobial peptides, or reactive oxygen species effectively destroy it as efficiently as the WT strain.

In summary, our study outlines the complex genetic pathways taken by *A. fumigatus* during evolution under increasing levels of Cu. We identify and analyze the novel genes *pma1*, *gcs1,* and *cpa1* that confer Cu resistance upon mutation. Our findings contribute to the identification of novel mechanisms of copper resistance in *A. fumigatus* and pave the way for future research directions in this field.

## Data Availability

All whole-genome sequences are available at SRA accession number PRJNA1094460.

## SUPPLEMENTAL MATERIAL

Supplemental material msphere.00253-24-s0001.docx Supplemental tables and figures.

ASM does not own the copyrights to Supplemental Material that may be linked to, or accessed through, an article. The authors have granted ASM a non-exclusive, world-wide license to publish the Supplemental Material files. Please contact the corresponding author directly for reuse.
