# Supplementary material for: Evolution of the pathogenic mold Aspergillus fumigatus on high copper levels identifies novel resistance genes
Source: mSphere. 2024 May 30;9(6):e00253-24. doi: 10.1128/msphere.00253-24 (PMC11332344; doi:10.1128/msphere.00253-24)
Supplement: Supplemental material — Supplemental tables and figures. [file msphere.00253-24-s0001.docx]

Supplementary Materials.

**Table S1**. Strains used in this study

| Strain | Genotype | Source |
| --- | --- | --- |
| CEA10 A1163 |  | d'Enfert, 1996 |
| AkuB^KU80^ | *KU80*∆ | Da Silva Ferreira et al., 2006 |
| WT30 |  | This study |
| WT A29 | *gcs1* FD481-482del ; *cpa1* A37V; AFUB_065670/*prx1* D104G; AFUB_016190/*ysh1* V162A | This study |
| WT B23 | *pma1* L424I ; *gcs1* FD481-482del ; *cpa1* A37V | This study |
| WT C30 | *pma1* L424I ; *gcs1* FD481-482del ; *cpa1* A37V AFUB_021700/*hse1* G326Stop; AFUB_081890/*myo1* D820N | This study |
| WT D30 | *pma1* L424I ; *gcs1* FD481-482del ; *cpa1* A37V; AFUB_069070 L101P | This study |
| ΔaceA6 | *KU80*∆ ; *aceA*∆ | This study |
| ΔaceA B22 | *KU80*∆ ; *aceA*∆ ; *pma1* V186del; AFUB_095920/E2 ubiquitin conjugating K9N | This study |
| ΔaceA C22 | *KU80*∆ ; *aceA*∆; *pma1* A414G; AFUB_071830 Y331stop, AFUB_088200/PIB1 Ubiquitin ligase G192R | This study |
| ΔaceA D22 | *KU80*∆ ; *aceA*∆; AFUB_024580/SltB L77F, AFUB_035240/*psd2* Q597stop, AFUB_021070/*png1* F186S, AFUB_078980 S251L | This study |
| ΔcrpA23 | *KU80*∆ ; *crpA*∆; | This study |
| ΔcrpA B23 | *KU80*∆ ; *crpA*∆ ; *pma1* V186del ; *ctrB* T142M; AFUB_034400 R464K, AFUB_055940/*ssk1* R690W, AFUB_059540/*pkcA* R50W, AFUB_081980/*coaT* I255N | This study |
| ΔcrpA C10 | *KU80*∆ ; *crpA*∆; *pma1* N158D ; *ctrB* T142M; AFUB_011550/*dscA* D528G, AFUB_042970 S515P, AFUB_090650 D528G | This study |
| ΔcrpA D15 | *KU80*∆ ; *crpA*∆; *pma1* L176P ; *ctrB* T142M | This study |
| Pma1 | *KU80*∆ ; *pma1* L424I | This study |
| Gcs1 | *KU80*∆ ; *gcs1* FD481-482del | This study |
| Cpa1 | *KU80*∆ ; *cpa1* A37V/*hph* | This study |
| Cpa1 WT | *KU80*∆ ; *cpa1* WT/*hph* | This study |
| Pma1-Gcs1 | *KU80*∆ ; *pma1* L424I ; *gcs1* FD481-482del | This study |
| Pma1-Cpa1 | *KU80*∆ ; *pma1* L424I ; *cpa1* A37V/*hph* | This study |
| Gcs1-Cpa1 | *KU80*∆ ; *gcs1* FD481-482del ; *cpa1* A37V/*hph* | This study |
| Triple | *KU80*∆ ; *pma1* L424I ; *gcs1* FD481-482del ; *cpa1* A37V/hph | This study |

**Table S2**. Amplification primers used in this study

| Target gene | Primer name | Primer sequence (5' → 3') | Final construct |
| --- | --- | --- | --- |
| Pma1 | Pma1 F | GGGCGTACCGTTAAGCACAAAG | Mutated Pma1 L424I |
|  | Pma1 R | TTACTCATCATCCTCCTCCACCTC |  |
| Gcs1 | Gcs F | CTGGCTATCTGGAACAGGGCCAA | Mutated Gcs1 FD481-482del |
|  | Gcs R | TTCGACAAGTACTGCGTCAAGGC |  |
| Cpa1 | Cpa1 F | GCATTGATTCGCTGTACGTAGCAAT | Mutated Cpa1 A37V |
|  | Cpa1-hph R | ATTGGACTTCTGTACCTAGGCTAGCTGGTCGCTTACCCAC |  |
| hph | Cpa1-hph F | GTGGGTAAGCGACCAGCTAGCCTAGGTACAGAAGTCCAAT | Hygromycin resistance cassette |
|  | Hph-Cpa1 3'Term R | GAACGAATCCGAGATTGAGGAATTTTCAGTAGCCTGGAGACAAATGAAGTTCTAGAAAGAAGGATTACCT |  |

**Table S3**. crRNAs used in this study

| Target gene | 5' crRNA | 3' crRNA |
| --- | --- | --- |
| Pma1 | GACGTCGTCGCCAGTCTTAA | GTTCTTGACGTTGTGGTTGG |
| Gcs1 | AAGAGCTTGGCGAACTTGTA | AACGGCTCATGCACGCAATG |
| Cpa1 | ACAGTAGCCATGAAGCGAGC | ATACTAGTGAAGGGCGGCAG |

**Table S4**. ARMP-PCR primers used in this study

| Target gene | Primer name | Primer sequence (5' → 3') |
| --- | --- | --- |
| Pma1 | Pma424 OF | ACGGCAATCTTGCTCAATGTTG |
|  | Pma424 WT R | GAGTACCAGTCTTGTCAGAGCAAAG |
|  | Pma424 Mut. F | TTGCTGGTGTCGACACCA |
|  | Pma424 OR | CCTTGTCCAGGTCATGATAGAG |
| Gcs1 | Gcs1 OF | GTTAGCGACTCATTTTGCCCAC |
|  | Gcs1 WT F | CGTGCCATTCTCAGCTTCGA |
|  | Gcs1 Mut. R | TCGGTATGTAGAAGTTGAGGCT |
|  | Gcs1 OR | TCCAGAGGGTTCCGTTTGCT |
| Cpa1 | Cpa1 OF | CGCCCCCTGCAATTATCATTTCTT |
|  | Cpa1 WT R | TCGTGAAAGTGGCACGCTCATTAG |
|  | Cpa1 Mut. F | TTCGTCAGCAGCGTCCGGT |
|  | Cpa1 OR | ACAGCAGTCCAGTGGCTGTA |

**Table S5**. Gene deletions and duplications identified in the Cu-evolved strains

| Strain | Deletions/Duplications |
| --- | --- |
| WT A29 | AFUB_004060 (del),AFUB_097890 (dupl), AFUB_097910 (dupl), AFUB_049930-AFUB_050800 (dupl) |
| WT B23 | AFUB_075250-AFUB_075430 (del) |
| WT C30 | AFUB_076890-AFUB_076950 (del) |
| WT D30 | AFUB_070960 (del), AFUB_071460 (del) |
| ΔaceA B22 | AFUB_063410-AFUB_063690 (del), AFUB_092930-AFUB_093120 (del), |
| ΔaceA C22 | AFUB_063130-AFUB_063370 (del), AFUB_063400-AFUB_063690 (del), AFUB_063740-AFUB_064180 (del), AFUB_072220-AFUB_072240 (del), AFUB_092930-AFUB_093120 (del) |
| ΔaceA D22 | AFUB_017350-AFUB_017690 (del), AFUB_034100-AFUB_034120 (del), AFUB_072930-AFUB_072930 (del), AFUB_072940-AFUB_073130 (del), AFUB_093150-AFUB_093160 (dupl) |
| ΔcrpA B23 | AFUB_062920-AFUB_063110 (dupl) |
| ΔcrpA C10 | AFUB_000010-AFUB_000140 (dupl), AFUB_017210-AFUB_017260 (dupl), AFUB_033870-AFUB_034380 (dupl), AFUB_048730-AFUB_048830 (dupl), AFUB_073130-AFUB_073130 (dupl), AFUB_080060-AFUB_080080 (dupl), AFUB_086620-AFUB_086660 (dupl) |
| ΔcrpA D15 | AFUB_000010-AFUB_000140 (dupl), AFUB_017210-AFUB_017260 (dupl), AFUB_033870-AFUB_034380 (dupl), AFUB_048730-AFUB_048830 (dupl), AFUB_073050-AFUB_073120 (deln), AFUB_073130-AFUB_073130 (dupl), AFUB_080060-AFUB_080080 (dupl), AFUB_086620-AFUB_086660 (dupl) |

**Construction and verification of the *gcs1* FD481-482del mutant strain.** Primers "Gcs1 F" and "Gcs1 R" (Table S2) were used for amplification of the mutated *gcs1* gene, with 50 bp flanking the 3' and 5' ends of the gene (Figure S1.A). The amplicon was introduced into *ΔKU80* strain, along with two gRNAs, one for each 5' and 3' ends of the target gene (Table S3), pTEL-hph and Cas9 enzyme (IDT). Protoplasts were plated on YPGS+ 250 µg/ml hygromycin. Transformant colonies were screened on YAG + 5 mM Cu agar plates, after which selected colonies were streaked twice on YAG agar plates for strain purification. Mutants were verified by AS-PCR with primers set to amplify the gene (primers "Gcs1 OF" and "Gcs1 OR"), the WT sequence when present ("Gcs1 WT F" and "Gcs1 OR") or the mutated sequence when present ("Gcs1 Mut. R" and "Gcs1 OF") (Table S4 and Figure S1.B). The final isolates displaying the correct band pattern were then verified by sequencing (Figure S1.C).


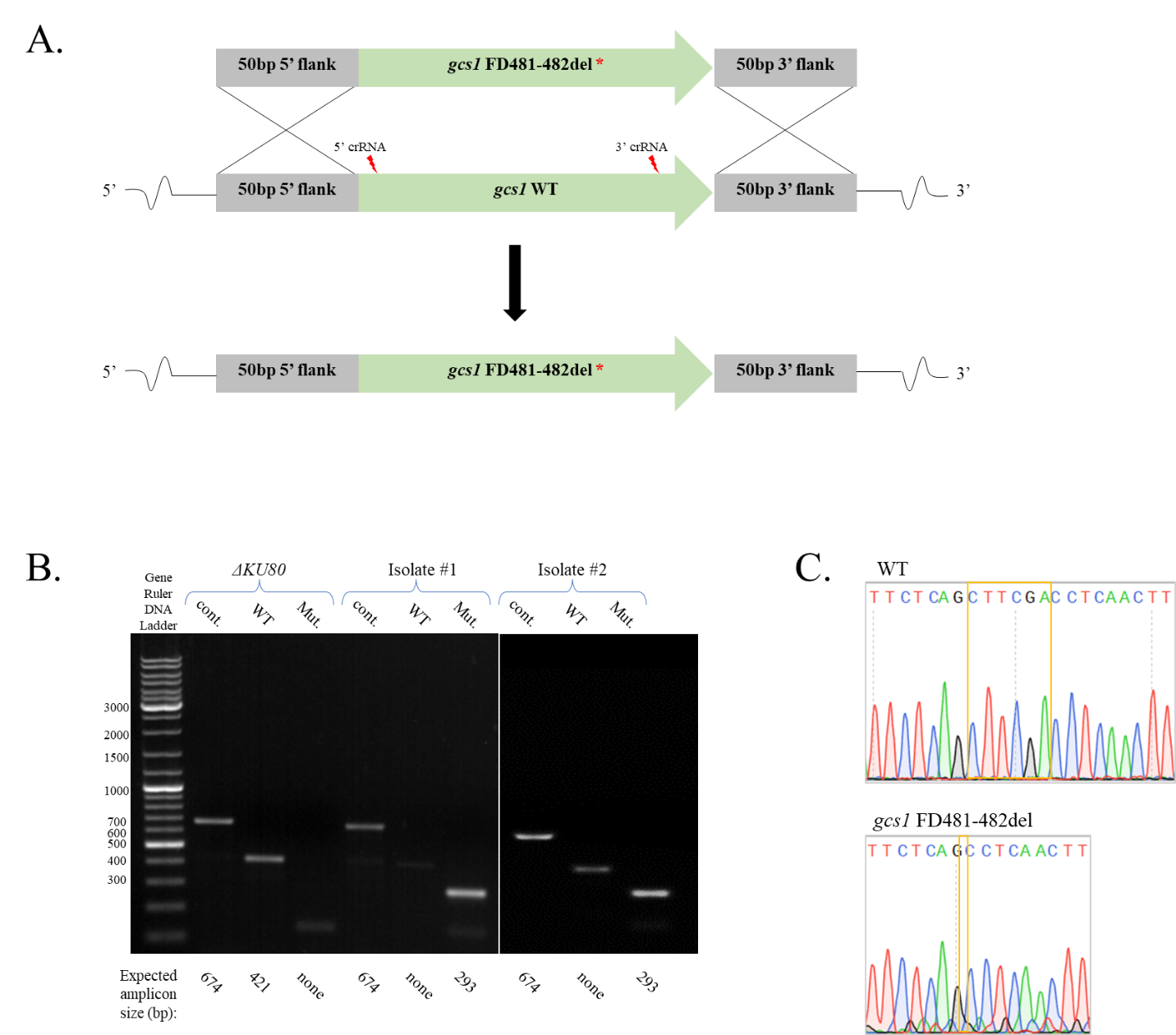


**Figure S1.** Generation and verification of *gcs1* FD481-482del mutant strain.

A. Schematic of gene replacement design. To introduce the *gcs1* mutation, the PCR-amplified mutated *gcs1* gene was used as a repair template, transformation was performed with pTel-hyg^R^ and two guide crRNAs *gcs1* 5’ and *gcs1* 3'. Similar methodology was used to generate all *gcs1* gene replacements described. B. ARMS-PCR analysis of one negative control isolate ("*ΔKU80*"), showing the control and "WT" bands, but not the "Mutant" band, and two isolates transformed with *gcs1* FD481-482del repair template, showing the control and "Mutant" bands, as well as a low intensity "WT" band. C. Strains were subsequently verified by Sanger sequencing.

**Construction and verification of the *pma1* L424I mutant strain.** Primers "Pma1 F" and " Pma1 R" (Table S2) were used for amplification of the mutated *pma1* gene, with 50 bp flanking the 3' and 5' ends of the gene (Figure S2.A). The amplicon was introduced into *ΔKU80* strain, along with two gRNAs, one for each 5' and 3' ends of the target gene (Table S3), pTEL-hph and Cas9 enzyme (IDT). Protoplasts were plated on YPGS+ 250 µg/ml hygromycin. Transformant colonies were screened on YAG + 5mM Cu agar plates, after which selected colonies were streaked twice on YAG agar plates for strain purification. Mutants were verified by AS-PCR with primers set to amplify the gene (primers "Pma1 OF" and "Pma1 OR"), the WT sequence when present ("Pma1 WT R" and "Pma1 OF") or the mutated sequence when present ("Pma1 Mut. F" and "Pma1 OR") (Table S4 and Figure S2.B). The final isolates displaying the correct band pattern were then verified by sequencing (Figure S2.C).


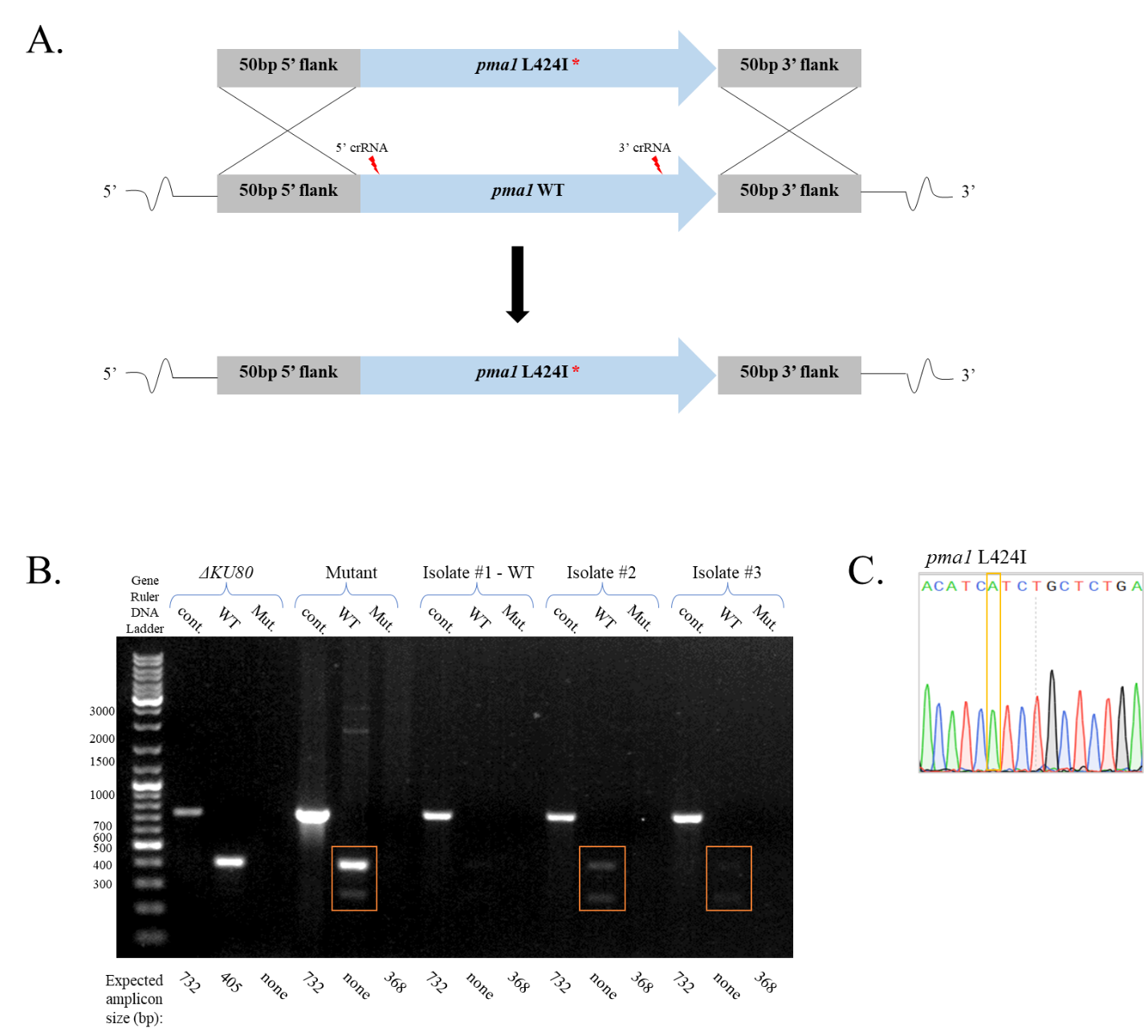


**Figure S2.** Generation and verification of *pma1* L424I mutant strain.

A. Schematic of gene replacement design. To introduce the *pma1* mutation, the PCR-amplified mutated *pma1* gene was used as a repair template, transformation was performed with pTel-hyg^R^ and two guide crRNAs *pma1* 5’ and *pma1* 3'. Similar methodology was used to generate all *pma1* gene replacements described. B. ARMS-PCR analysis of one negative control isolate ("*ΔKU80*"), one positive control isolate ("Mutant"), and three isolates transformed with *pma1* L424I repair template. All isolates display the control band. As the "mutant" primer pair did not yield a band in the positive control isolate, suspectedly transformed isolates were selected based on the double band pattern in the "WT" lane, boxed in orange. C. Strains were subsequently verified by Sanger sequencing.

**Construction and verification of the *cpa1* A37V mutant strain.** Initial efforts to generate the *cpa1* A37V mutant strain under Cu selection using the PCR-amplified mutated *cpa1* gene as a repair template failed. Therefore, a conventional approach was taken, generating a mutated *cpa1* gene fused to the *hph* resistance cassette as a repair template. Primers "Cpa1 F" and "Cpa1-hph R" (Table S2) were used for amplification of the mutated *cpa1* gene, with 50 bp flanking the 5' end of the gene, primers "Cpa1-hph F" and " Hph-Cpa1 3'Term R " were used for amplification of the hph resistance cassette, with 50 bp flanking the 3' end of the *cpa1* gene (Figure S3.A). The amplicons were fused together using the overlap PCR method, and the final construct was introduced into the *ΔKU80* strain, along with two gRNAs, one for each 5' and 3' ends of the target gene (Table S3), and Cas9 enzyme (IDT). Transformant colonies were screened on YPGS + hygromycin (200 μg/ml) agar plates, after which selected colonies were streaked twice on YAG agar plates for strain purification. Mutants were verified by AS-PCR with primers set to amplify the gene (primers "Cpa1 OF" and "Cpa1 OR"), the WT sequence when present ("Cpa1 WT R" and "Cpa1 OF") or the mutated sequence when present ("Cpa1 Mut. F" and "Cpa1 OR") (Table S4 and Figure S3.B). The final isolates displaying the correct band pattern were then verified by sequencing (Figure S3.C).


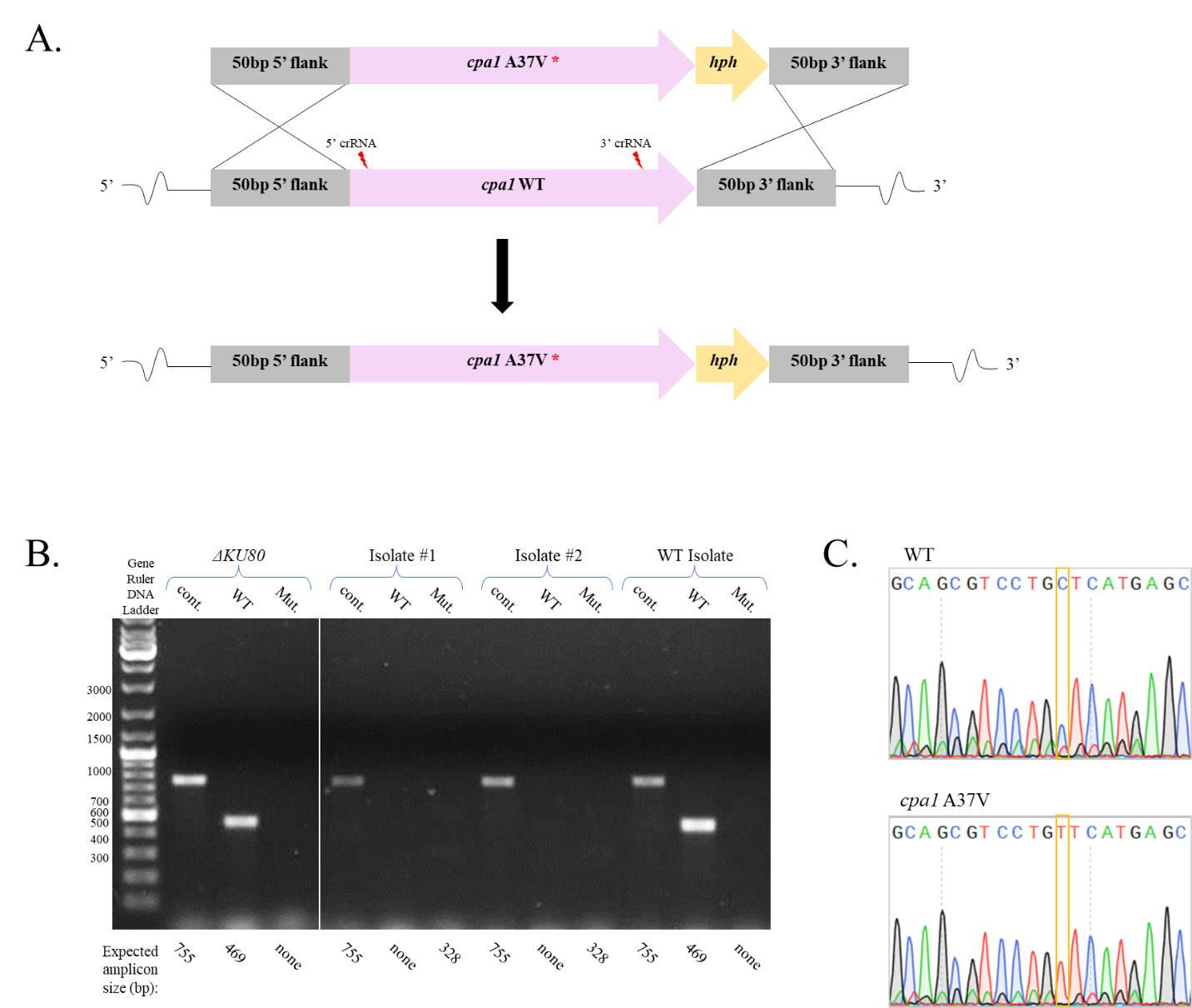


**Figure S3.** Generation and verification of *cpa1* A37V mutant strain.

A. Schematic of gene replacement design. To introduce the *cpa1* mutation, the PCR-amplified mutated or WT *cpa1* gene and the hygromycin resistance cassette were fused together using the overlap PCR method, and the final product was used as a repair template, transformation was performed with two guide crRNAs *cpa1* 5’ and *cpa1* 3'. Similar methodology was used to generate all *cpa1* gene replacements described. B. ARMS-PCR analysis of one negative control isolate ("*ΔKU80*"), two isolates transformed with *cpa1* A37V/ hph repair template, and one isolate transformed with *cpa1* WT/ hph repair template ("WT isolate"). All isolates display the control band, the negative control isolate and the WT isolate both display the "WT" band but not the "Mutant" band, while both *cpa1*-mutated isolates display the "Mutant" band, but not the "WT" band. C. Strains were subsequently verified by Sanger sequencing.

**Construction and verification of the *gcs1* FD481-482del / *cpa1* A37V double mutant strain.** The *gcs1 FD481-482del* mutant strain was used as a background strain for the introduction of the *cpa1* A37V mutation. The *cpa1* A37V mutation was introduced as described above, selected on YPGS + hygromycin (200 μg/ml) agar plates, screened for *cpa1* and *gcs1* mutations using AS-PCR, and sequence-verified.

**Construction and verification of the *pma1* L424I / *cpa1* A37V double mutant strain.** The *pma1* L424I mutant strain was used as a background strain for the introduction of the *cpa1* A37V mutation. The *cpa1* A37V mutation was introduced as described above, selected on YPGS + hygromycin (200 μg/ml) agar plates, screened for *cpa1* and *pma1* mutations using AS-PCR, and sequence-verified.

**Construction and verification of the *gcs1* FD481-482del / *pma1* L424I double mutant strain.** The *gcs1 FD481-482del* mutant strain was used as a background strain for the introduction of the *pma1* L424I mutation. The *pma1* L424I mutation was introduced as described above, selected on YAG + 6 mM Cu agar plates, screened for *gcs1* and *pma1* mutations using AS-PCR, and sequence-verified.

**Construction and verification of the *gcs1* FD481-482del / *pma1* L424I / *cpa1* A37V triple mutant strain.** The *gcs1* FD481-482del / *pma1* L424I double mutant strain was used as a background strain for the introduction of the *cpa1* A37V mutation. The *cpa1* A37V mutation was introduced as described above, selected on YPGS + hygromycin (200 μg/ml) agar plates, screened for *cpa1*, *gcs1* and *pma1* mutations using AS-PCR, and sequence-verified.

**Figure S4**. **Radial growth of the reconstituted strains under stress**. Strains were point inoculated in quadruplicate on YAG agar plates under the various stress conditions indicated. Radial colony size was measured after 3 days at 37C. There were no statistically significant differences in radial growth between the strains.


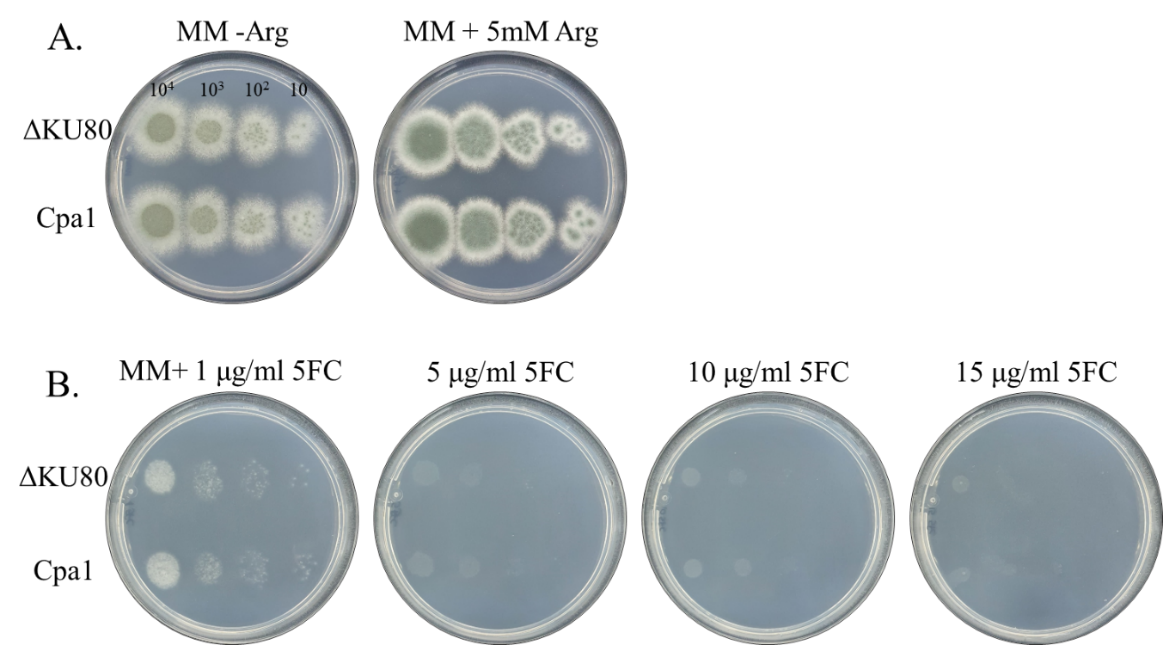


**Figure S5**. **Growth of the WT (ΔKU80) and Cpa1 mutant strain in the absence of arginine and in the presence of increasing concentrations of 5-flucytosine (5FC).** Strains were point inoculated on MM plates (10^4^-10 CFU/point inoculation), and grown for 48 h at 37°C, (A) -/+ arginine supplementation and (B) in the presence of increasing concentrations of 5FC.

**Figure S6. Determination of total glutathione GSH/GSSG concentrations in WT (ΔKU80) and Gcs1 strain with and without Cu**. Strains were grown overnight in liquid YAG at 37°C, 4 mM Cu was added to +Cu mycelium for 1 h. Mycelium was flash frozen in liquid nitrogen and lyophilized. Determination of glutathione (GSH/GSSG) concentrations was performed using the glutathione colorimetric detection kit (Arbor Assays, MI, USA), according to the protocol provided. There are no statistically significant differences between strains WT/Gcs1 or WT+Cu/Gcs1+Cu.
